# Proteochemometric modeling in a Bayesian framework

**DOI:** 10.1186/1758-2946-6-35

**Published:** 2014-06-28

**Authors:** Isidro Cortes-Ciriano, Gerard JP van Westen, Eelke Bart Lenselink, Daniel S Murrell, Andreas Bender, Thérèse Malliavin

**Affiliations:** 1 Institut Pasteur, Unité de Bioinformatique Structurale; CNRS UMR 3825; Département de Biologie Structurale et Chimie; 2 ChEMBL Group, European Molecular Biology Laboratory European Bioinformatics Institute, Wellcome Trust Genome Campus, CB10 1SD, Hinxton, Cambridge, UK; 3 Division of Medicinal Chemistry, Leiden Academic Center for Drug Research, Leiden, The Netherlands; 4 Unilever Centre for Molecular Science Informatics, Department of Chemistry, University of Cambridge, Cambridge, UK

**Keywords:** Proteochemometrics, Bayesian inference, Gaussian process, Chemogenomics, GPCRs, Adenosine receptors, Applicability domain

## Abstract

Proteochemometrics (PCM) is an approach for bioactivity predictive modeling which models the relationship between protein and chemical information. Gaussian Processes (GP), based on Bayesian inference, provide the most objective estimation of the uncertainty of the predictions, thus permitting the evaluation of the applicability domain (AD) of the model. Furthermore, the experimental error on bioactivity measurements can be used as input for this probabilistic model.

In this study, we apply GP implemented with a panel of kernels on three various (and multispecies) PCM datasets. The first dataset consisted of information from 8 human and rat adenosine receptors with 10,999 small molecule ligands and their binding affinity. The second consisted of the catalytic activity of four dengue virus NS3 proteases on 56 small peptides. Finally, we have gathered bioactivity information of small molecule ligands on 91 aminergic GPCRs from 9 different species, leading to a dataset of 24,593 datapoints with a matrix completeness of only 2.43%.

GP models trained on these datasets are statistically sound, at the same level of statistical significance as Support Vector Machines (SVM), with $R_{0}^{2}$ values on the external dataset ranging from 0.68 to 0.92, and RMSEP values close to the experimental error. Furthermore, the best GP models obtained with the normalized polynomial and radial kernels provide intervals of confidence for the predictions in agreement with the cumulative Gaussian distribution. GP models were also interpreted on the basis of individual targets and of ligand descriptors. In the dengue dataset, the model interpretation in terms of the amino-acid positions in the tetra-peptide ligands gave biologically meaningful results.

## Background

The advent of high-throughput (HT) technologies has contributed in the last decades to a vast data increase in proprietary and public bioactivity databases. In a parallel manner, a large amount of biological data has been collected on protein structure and sequence information for numerous species. Chemogenomic techniques [1-3] can capitalize on this large amount of information by modeling the relationships between the chemical and the biological space. This data integration permits the bioactivity prediction of compound-target combinations lying in regions of the drug-target interaction space which are sparsely sampled by experimental measurements. These techniques are based on the similarity principle [4,5], which follows the premise that similar compounds (and targets) [6] are more likely to exhibit akin bioactivity profiles in comparison to structurally distant structures. Among others, chemogenomic approaches have enabled: (i) the prediction of protein targets for new compounds based on the bioactivity profiles of similar compounds, [7-9] (ii) the study of protein similarity on the basis of the similarity of their ligands, [10,11] and (iii) receptor deorphanization [12].

In the field of chemogenomics, Proteochemometrics (PCM) [6] uses machine learning models to relate compounds to their biomolecular targets (usually proteins). PCM extends traditional Quantitative Structure-Activity Relationship (QSAR) [13] by allowing to both inter- and extrapolate on the target and/or chemical spaces. Therefore, compounds can be optimized not only with respect to their affinity on a target, [14] but also by taking into account their selectivity [15]. In that way, PCM also permits to detect compound substructures conferring inhibitory activity to a panel of related biomolecular targets [14].

Although the relevance of PCM has been confirmed by both *in silico* and experimental validation, [6,16] current methods cannot: (i) inherently determine the applicability domain (AD) of a model, or (ii) provide individual confidence intervals for each prediction.

The applicability domain (AD) of a bioactivity model is defined as the range of chemical (and target in PCM) space to which the model can be reliably applied [17-19]. Therefore, the AD is a measure of the generalization properties of a given model: the volume of chemical (descriptor) space that can be reliably predicted [20]. Given that compounds are encoded with descriptors when training predictive models, it is important to distinguish between the chemical space (referring to chemical structures) and the chemical descriptor space. This distinction is important as in the calculation of some popular descriptors (*e.g.* Morgan fingerprints) [21], chemical substructures are hashed: different chemical substructures are mapped at the same descriptor position. Consequently, two different structures in the chemical space can be represented by the same descriptor values. A detailed discussion of the different methods proposed to assess models AD can be found in Ref., [20] to which the interested reader is referred. In PCM, the AD is an essential feature, as extrapolation has to be used to predict the bioactivity for *new* chemicals on *new* targets [6].

In parallel to the concern about the evaluation of individual bioactivity predictions, recent publications have aimed at establishing the level of uncertainty in public bioactivity databases [22-25]. In this vein, Brown *et al.*[26] highlighted the importance of including the uncertainty of bioactivity data into the evaluation of models quality. Hence, predictive models should be assessed through: the analysis of the experimental error of the data, the evaluation of the models AD as well as the definition of intervals of confidence for the predictions. However, acceptable levels of prediction errors are also determined by the context in which the model will be applied. Indeed, models exhibiting high prediction errors can be nevertheless useful in a high-throughput (HTS) campaign while not being suitable in lead optimization [26].

Bayesian inference provides a reliable theoretical framework to handle all previously mentioned aspects within a unique bioactivity model. Gaussian Processes (GP) are a non-parametric machine learning method based upon Bayesian inference: they thus permit an evaluation of the AD of a given model as well as providing the most objective estimation of the predictions uncertainty. Furthermore, the experimental bioactivity errors can be used as model input. A GP prediction of a given compound-target combination is a Gaussian distribution whose variance defines intervals of confidence: in principle, this variance measures the distance of the compound-target pair to the training set. GP models can be globally validated by traditional statistical metrics (*e.g.R*^2^ or *Q*^2^) [27-29] while also providing individual assessment for predictions. GP were firstly introduced in the field of QSAR modeling by Burden *et al.*[30]. Later on, GP were also used for: (i) the modeling of ADMET properties, [31,32] (ii) the prediction of electrolyte solubility, [33] (iii) the bioactivity prediction of small peptide datasets, [34-36] (iv) protein engineering, [37] and (v) the bioactivity prediction of bioactivity-focused (GPCRs) combinatorial chemolibraries [38]. The purpose of the present study is to propose Gaussian Process (GP) to simultaneously model chemical and multispecies protein information in the frame of PCM. GP models are validated by comparing their performance to that of SVM using a panel of kernels on two PCM datasets extracted from ChEMBL database, [39] involving adenosine receptors (10,999 data points, 8 sequences) and aminergic GPCRs (24,593 data points, 91 sequences), and on a third dataset extracted from the literature concerning the catalytic activity of four dengue virus NS3 proteases (199 data points, 4 sequences). GP perform as well as SVM, with statistically non-significant differences in performance. Nonetheless, GP provide additional information with respect to SVM, namely the uncertainties on individual bioactivity predictions. GP also permit the interpretation of the models with respect to the targets of adenosine receptors and GPCR datasets, and also with respect to the ligand descriptors.

## Methods

### Datasets

#### Aminergic GPCRs

The aminergic GPCRs dataset was assembled by gathering bioactivity information of 91 different receptors (9 species) from ChEMBL 15, [39] producing a total number of datapoints of 24,593. A high quality bioactivity dataset was assembled by keeping only assay-independent bioactivity information, namely: the constant of inhibition, *K*_*i*_, and the constant of dissociation, *K*_*d*_. In those cases where a given compound-target pair had multiple bioactivity values annotated, the mean value was used. Moreover, annotations with anything other than ‘=’ were discarded. Agonist, antagonist and partial agonist ligands were included. Bioactivity values in the dataset range from 2.030 to 11.570 *pK*_*i*_ units. The component amino acids of the transmembrane binding site were taken from Gloriam *et al.*[40] Further information about the dataset can be found in Table 1 and Additional file 1: Table S2.

**Table 1:** Overview of the proteochemometric datasets modeled in this work

	**Adenosine receptors**	**Dengue virus NS3 Proteases**	**Aminergic GPCRs**
Datapoints	10,999	199	24,593
Sequences	8	4	91
Ligands	4,419	56	11,121
Source Organisms	*H. sapiens and Rattus norvegicus*	*Dengue virus*	*H. sapiens, Rattus norvegicus, Mus musculus, Bos taurus, Sus scrofa, Canis familiaris, Cavia porcellus, Chlorocebus aethiops, and Mesocricetus auratus*
Bioactivity	*pK*_*i*_	*K*_*c* *a* *t*_	*pK*_*i*_
Matrix Completeness (%)	31.11	88.84	2.43

Whereas the compound-target interaction matrix of the dengue virus NS3 proteases dataset is almost complete (88.84%), the adenosine receptors and GPCRs dataset are more challenging to model given: (i) their sparsity (31.11 and 2.43% of matrix completness respectively), and (ii) the consideration of information from human orthologues, being the respective number of different sequences 8 and 91.

#### Adenosine receptors

This dataset previously published by van Westen *et al.*[16] is composed of 10,999 bioactivity data points measured on the rat and human adenosine receptors, *A*_1_, *A*_2*A*_, *A*_2*B*_ and *A*_3_. The dataset was extracted from ChEMBL 2. Only compounds tested on rat or human receptors by radio-ligand binding assays and for which *pK*_*i*_ bioactivity values were annotated with a ‘=’ relationship were included in the final dataset. Bioactivity values range from 4.50 to 10.52 *pK*_*i*_ units. Compounds were normalized and ionized at pH 7.4. Subsequently, they were assigned 2D coordinates and converted to fingerprints. See Table 1 for further details about the dataset.

#### Dengue virus NS3 proteases

This dataset was collected from the proteochemometric study published by Prusis *et al.*, [41] which modeled the catalytic activity of the Dengue virus NS3 proteases from four viral serotypes using datapoints measured on 56 different tetra-peptide substrates (Table 1). These substrates were designed to evaluate the role amino acid residues located at P1’-P4’ in the sequence. The catalytic efficiency was measured as the turnover number (*k*_*c* *a* *t*_) for the cleavage of the substrate. In contrast to the two datasets presented above, the number of data points in this case was only 199.

### Descriptors

Chemical compounds were described by Scitegic circular fingerprints (ECFP_6 type), [21,42] calculated in PipelinePilot 8.5.0.200 [43]. For the calculation of keyed ECFP_6 fingerprints, each compound substructure, with a maximal diameter of three bonds, is treated as a compound feature. The substructures are then mapped into an unhashed array of counts, thus enabling the estimation of their contribution to bioactivity (see Results and Discussion). The efficiency of these fingerprints to identify chemical features relevant for bioactivity has been previously demonstrated [16,44]. Pairwise compound similarity plots were calculated in R using the *vegan* package [45]. Protein amino acids of the GPCRs and adenosine receptors binding sites, as well as the Dengue virus NS3 proteases substrates, were described with five amino acid extended principal property scales (5 z-scales). The property calculation was conducted in R [46]*via* in-house scripts following the work of Sandberg *et al.*[47]. In the GPCRs dataset a descriptor accounting for the amino acids side chain charge at pH 7.4 was also added (with values of: +1 if the charge is positive, -1 if negative and 0 for neutral amino acids). The four Dengue virus NS3 protease variants were described with binary descriptors.

### Modeling with Bayesian inference

#### Gaussian processes

Given a dataset *D*={**X**,**y**} where $\text{X}={\left\{{\text{x}}^{i}\right\}}_{i=1}^{n}$ is the set of compound and target descriptors, and $\text{y}={\left\{y^{i}\right\}}_{i=1}^{n}$ is the vector of observed bioactivities, the aim is to find a Gaussian Process [48], *GP*(**D**), capable to infer the relationships within **D**, in order to predict the bioactivity *y*^⋆^ for new compound-target combinations **x**^⋆^. In the frame of Bayesian inference, GP are defined as:

(1) $$\begin{array}{l} P(\text{GP}(\text{D})|\text{D})\propto P(\text{y}|\text{GP}(\text{D}),\text{X})\;P(\text{GP}(\text{D})) \end{array}$$

where: (i) *P*(*GP*(**D**)|**D**) is the *posterior* probability distribution giving the bioactivity predictions, (ii) the likelihood *P*(**y**|*GP*(**D**),**X**) is the probability of the observations, **y**, given the training set, **X** and the model *GP*(**D**), and (iii) *P*(*GP*(**D**)) is the *prior* probability distribution of the functions *GP*(**D**) candidates to model the dataset **D**.

The *prior* probability distribution is updated with the information contained in **D** via the likelihood, leading to the definition of the *posterior* probability distribution as the set of functions efficiently modeling **D**. The average of the *posterior* distribution is considered as the bioactivity prediction (Additional file 1: Figure S1). *GP*(**D**) is a random function which functional values follow a centered Gaussian distribution for any set of datapoints. Thus, the *P*(*GP*(**D**)) values for a finite subset of compound-target vectors **x**_*i*_,..,**x**_*n*_ follow a multidimensional normal distribution with mean *μ* and covariance matrix **C**_***X***_:

(2) $$\begin{array}{l} \text{GP}(\text{D})∼N\left(0,\;C_{X}+\sigma_{d}^{2}\delta \left(x_{j},x_{k}\right)\right)\;\left(\text{j,k}\in 1,\ldots ,n\right) \end{array}$$

where *δ*(**x**_***j***_,**x**_***k***_) is the Kronecker delta function and $\sigma_{d}^{2}$ is the noise of the datapoints (experimental error), which is assumed to be normally distributed with mean zero. The value of $\sigma_{d}^{2}$ accounts for the noise in the observed bioactivities, $\text{y}=\text{GP}(\text{D})+N\left(0,\sigma_{d}^{2}\right)$ which in turn reflects the trade-off between the quality and smoothness of the fitting.

**C**_***X***_ is obtained by applying a positive definite kernel function (also known as *statistic* covariance) [49] to **X**,**C**_*X*_=*Cov*(**X**). Owing to the fact that the covariance function is based upon dot products, the *kernel trick* can be applied in a similar way as in SVM [50]. Kernel parameters are called hyperparameters since their values define the probability of each function of the *prior* probability distribution. The different kernels implemented in this study are listed in Additional file 1: Table S2.

#### Bioactivity prediction for new datapoints

The bioactivity, *y*^⋆^, of a new compound-target combination, **x**^⋆^, can be predicted from the joint prior probability distribution P=yy⋆ of *y* and *y*^⋆^, due to the multivariate Gaussian distribution assumed for **D**:

(3) $$\begin{array}{l} \;\left[\begin{array}{l} \text{y}\\ y^{⋆} \end{array}\right]\;∼\;N\;\left(\;\;\begin{array}{ll} 0, & \;\;C^{⋆}\;=\;\left[\;\begin{array}{ll} C_{X}=\text{Cov}(\text{X}), & k=\text{Cov}\left(X,x^{⋆}\right)\\ k^{T}, & m=\text{Cov}\left(x^{⋆},x^{⋆}\right) \end{array}\;\right] \end{array}\;\right) \end{array}$$

where *k*^*T*^ is the transpose of the matrix *k*, which describes the similarity between **X** and **x**^⋆^. The predicted bioactivity is obtained as the mean value of the probability:

(4) $$\begin{array}{l} P\left(y^{⋆}|x^{⋆},\text{D},\text{y}\right) \end{array}$$

and the uncertainty of the prediction corresponds to the standard deviation of this probability distribution.

To calculate *P*(*y*^⋆^|**x**^⋆^,**D**,**y**), the joint probability distribution, Pyy⋆, is divided by the probability of the observed bioactivities, *P*(**y**). Subsequently, the predicted probability for *y*^⋆^ is obtained by calculating the Schur complement [51]:

(5) $$\begin{array}{l} P(y^{⋆})∼N\left(\mu_{y^{⋆}}=k^{T}C_{X}^{-1}\text{y},\;\sigma_{y^{⋆}}^{2}=m-k^{T}C_{X}^{-1}k\right) \end{array}$$

where the best estimate for the bioactivity of **x**^⋆^ is the average value of *y*^⋆^, $\mu_{y^{⋆}}=\langle P(y^{⋆})\rangle$, and $\sigma_{y^{⋆}}$, the standard deviation, its uncertainty.

As can be seen in Eq. 5, those compound-target combinations in **X** similar to **x**^⋆^, contribute more to the prediction of *y*^⋆^, as **y** is weighted by *k*^*T*^. This means that GP, as a kernel method, mainly infers the value of *y*^⋆^ from the most similar compound-target combinations in descriptor space present in the training set, **X**.

On the other hand, the predicted variance, $\sigma_{y^{⋆}}^{2}$, is equal to the difference between the *a priori* knowledge about **x**^⋆^: *m*=*Cov*(**x**^⋆^,**x**^⋆^), and what can be inferred about **x**^⋆^ from similar compound-target combinations present in **X**: $k^{T}C_{X}^{-1}k$. Thus, in the case of **x**^⋆^ being similar to the compound-target combinations in **X**, the value of $\sigma_{y^{⋆}}^{2}$ is small. By contrast, a high value of $\sigma_{y^{⋆}}^{2}$ indicates that **x**^⋆^ is not similar (is distant) to the compound-target combinations in **X**. In that case, the GP cannot learn much about **x**^⋆^ from the training set, so the prediction should be consider as less reliable. Consequently, $\sigma_{y^{⋆}}^{2}$ gives an idea of the applicability domain (AD) of the model and thus serves to evaluate the uncertainty of the prediction.

### Computational details

#### Determining the kernel hyperparameters

As previously stated (Equation 2), the prior distribution of a GP is mainly defined by its covariance, **C**_*X*_, which is in turn characterized by its hyperparameter values. For the simplest kernel, Radial Basis function kernel (RBF), also known as Squared Exponential or simply Radial (Additional file 1: Table S1), the hyperparameters are $\left(\Omega =\left\{l,\sigma_{d}^{2}\right\}\right)$ where *l* are the length scales, (one per descriptor) and $\sigma_{d}^{2}$ the noise variance. In this case, the covariance between two input vectors can be defined as:

(6) $$\begin{array}{l} \text{Cov}\left(x_{i},x_{j}\right)=e^{-\frac{1}{2}\overset{P}{\underset{p=1}{\sum }}\frac{{\left(x_{p}^{i}-x_{p}^{j}\right)}^{2}}{l_{p}^{2}}} \end{array}$$

where p is the descriptor index and P the total number of descriptors. Each length scale, *l*, is treated as a hyperparameter wich value needs to be optimized during model training. High length scale values will be assigned to irrelevant features for the model. Therefore, the inverse of the optimized *l* value obtained for a given descriptor gives an idea of its importance for the model. This inherent ability of Bayesian inference to infer the relevance of each descriptor is the so-called Automatic Relevance Determination (ARD) [48]. In the context of PCM, ARD can be exploited to provide a biologically meaningful interpretation of the models.

In the frame of Bayesian inference, we search for the hyperparameter values maximizing the probability of having obtained the observed data. Thus, the hyperparameter values should define a prior distribution *P*(*GP*(**D**)) maximizing the probability of the functions along the data. The problem can be rewritten as: the search of hyperparameter values maximizing the posterior probability distribution over the hyperparameters: *P*(*Ω*|**D**). In a Bayesian line of reasoning, this posterior probability can be expressed as:

(7) $$\begin{array}{l} P(\Omega |\text{D})∼P(\text{y}|\Omega ,\text{X})\;P(\Omega ) \end{array}$$

where *P*(**y**|*Ω*,**X**), is the marginal likelihood: P(y|Ω,X)=∫P(y|GP(D)P(GP(D))dGP(D). The hyperparameter values *Ω* can thus be determined by maximizing the logarithm of the marginal likelihood [48,52]:

(8) $$\begin{array}{l} \ln P(\text{y}|\Omega ,\text{X})=-\frac{1}{2}{\text{y}}^{T}C^{-1}\text{y}-\frac{1}{2}\ln|C|-\frac{\text{n}}{2}\ln2\pi \end{array}$$

Several methods can be implemented to accomplish this multivariate optimization problem, such as a simplex method, Monte Carlo (MC) Sampling, [53] a genetic algorithm, nested sampling, [54] forward variable selection [31] or the conjugate gradient method [48].

In the present study, kernel hyperparameters were optimized by grid search and *k*-fold cross-validation (CV) in the case of the adenosine receptors and aminergic GPCRs datasets (section S1 of the Additional file 1), because of their large size and high number of descriptors. The experimental error, $\sigma_{d}^{2}$, (Equation 2) was considered as fixed with a value of 0.29 *pK*_*i*_ units, this value being taken from the work of Kramer *et al.*[22] The same length scale value, *l*, was used for all descriptors to simplify the hyperparameter optimization.

In the case of the dengue virus dataset, due to its small size, and to the lack of information concerning the experimental uncertainty, the noise variance, $\sigma_{d}^{2}$, was optimized by conjugate gradient as implemented in the GPML toolbox [55]. As the number of descriptors is only 24, we optimized the length scales using the radial kernel. In the frame of Automatic Relevance Determination (ARD), the importance of each descriptor for the model was estimated using the inverse of the optimized *l* values, in the way described above.

#### GP Tolerance to noise

To better understand the influence of the experimental error in GP modeling, we trained 15 models for each dataset with increasing levels of noise with both the radial and the normalized polynomial (NP) kernel, thus leading to a total number of 90 models. Their predictive ability was monitored on the external set. The levels of added noise (noise variance) ranged from 0 to a maximum value of 10, which corresponds to a noise deviation of 3.2 *pK*_*i*_ units for the adenosine receptors and GPCR datasets, and 3.2 log units for the dengue virus NS3 proteases dataset.

#### Machine learning analyses and implementation

Machine learning models were built in R using the *caret* package [56]. Non-default kernels for GP were introduced in the caret framework by in-house R scripts and by the definition of custom models (*custom* option in the *caret* package) implementing kernel functions from either the *kernlab*[57] package or in-house kernel functions. Source code is available from the authors upon request. Likewise, The Gaussian Process for Machine Learning (GPML) Toolbox version 3.2 [55] was used to build GP models in Matlab version 7.15 [58] to assess the importance of ligand descriptors (Automatic Relevance Determination). The python package *infpy*[59] helped to generate Additional file 1: Figure S1. The data pre-processing and the *in silico* modeling pipeline are described in Additional file 1, along with model training and validation.

### Assessment of maximum model performance

The Tropsha validation criteria, [27-29] (Equations S7-S10 in Additional file 1) were used for accepting or dismissing the model (section Internal validation of Additional file 1). Hence, the distributions of minimum RMSEP_ext_ and maximum, $Q_{\text{ext}}^{2}$, $R_{0\;\text{ext}}^{2}$, and $R_{\text{ext}}^{2}$ (Equations S3-S6 in Additional file 1) were calculated for each dataset in the following way. Firstly, a random sample, *A*, of the same size of the external set was drawn from the experimental bioactivity values. Secondly, the sample *B* was calculated by adding to *A* a random noise with mean zero and standard deviation equal to the experimental error. Then, the statistical metrics were calculated for *A* with respect to *B*. The calculation of statistical metrics on 1,000 generations of random samples *A* and noisy samples *B* provided a distribution of statistical metrics for each dataset. These maximum and minimum values of the distribution were then used to validate the metrics values obtained when evaluating the bioactivities predicted for the external sets. If the obtained metrics were beyond the maximum values (for $Q_{\text{ext}}^{2}$, $R_{0\;\text{ext}}^{2}$, and $R_{\text{ext}}^{2}$) or the minimum values (for RMSEP_ext_) of the distribution, the model is likely to be over-optimistic. The experimental errors required to define the random samples *B* were determined in the following way. For adenosine and GPCR datasets, the experimental error of *pK*_*i*_ data was considered to be approximately 0.29 *pK*_*i*_ units, which corresponds to the average standard deviation value for public *K*_*i*_ datasets estimated by Kramer *et al.*[22] The experimental error of the dengue dataset was inferred from the data by considering its uncertainty as a hyperparameter of the GP model since we could not find information about the experimental uncertainty in the study of Prusis *et al.*[41].

### Interpretation of ligand substructures

To calculate the influence of a given feature (chemical substructure) to *pK*_*i*_, we iteratively set the count of the feature equal to zero in all compound descriptors presenting it, in order to virtually remove the substructure. Bioactivity values were then predicted using the modified compound descriptors, and the difference between the predicted values in the presence or absence of a given feature were calculated. The average value of these differences, weighted by the number of counts of the feature in each compound, corresponds to the contribution of that feature to bioactivity. The contribution was estimated for all compound features considered in the model. The sign of the difference ({+/-}) indicates if the feature is respectively beneficial or deleterious for compound bioactivity. This approach is closely related to the method proposed by van Westen *et al.*, [14] although two modifications have been made: (i) the weighting of the average difference between predicted and observed bioactivities, and (ii) the calculation of descriptor importance on a per target basis.

## Results

### Model validation

#### PCM GP models agree with the validation criteria

Overall, the models obtained for the three datasets with Gaussian Process modeling display statistics in agreement with our validation criteria (Table 2 and Additional file 1: Table S3). To ensure that these results were not the consequence of spurious correlations, we trained GP models with randomized bioactivity values (y-scrambling) [60]. For all datasets, the intercept was negative, thus ensuring the statistical soundness of our modeling. The best GP model for the adenosine receptors dataset was obtained with the normalized polynomial (NP) kernel, exhibiting RMSEP_ext_ and $R_{0\;\text{ext}}^{2}$ values of 0.58 *pK*_*i*_ units and 0.75 respectively. Similarly, in the case of the GPCRs dataset, the NP kernel led to the best predictive model, with RMSEP_ext_ and $R_{0\;\text{ext}}^{2}$ values of 0.66 *pK*_*i*_ units and 0.72. As these GP models were trained with a noise deviation of 0.54 *pK*_*i*_ units, the subtraction of the experimental uncertainty, 0.54 *pK*_*i*_ units, from the RMSEP_ext_ gives a residual error arising from the modeling below 0.12 *pK*_*i*_ units. These RMSEP_ext_ values correspond to 6.05% and 10.88% of the range of bioactivity values in the training set for the GPCRs and the adenosine receptors datasets. In the case of the dengue virus dataset, GP models show better predictive ability than those reported by Prusis *et al.*, [41] as $Q_{\text{ext}}^{2}$ value of 0.92 is obtained here (Additional file 1: Table S3) for the best GP model based on the Bessel kernel. The optimization of the noise variance, $\sigma_{d}^{2}$, as an hyperparameter during the training process led to a value of 0.27 log units, similar to the values of about 0.3 log units reported by Prusis *et al.*[61] in a recent study with similar experimental setup.

**Table 2:** Internal and external validation metrics for the PCM models

		**Adenosine Receptors Dataset**		
	$R_{\text{int}}^{2}$	**RMSEP**_**int**_	$R_{0\;\text{ext}}^{2}$	**RMSEP**_**ext**_
GP Bessel	0.64	0.70	0.70	0.63
GP Laplacian	0.67	0.68	0.67	0.66
GP Norm. Polynomial (NP)	0.69	0.65	0.75	0.58
GP Polynomial	0.70	0.64	0.70	0.63
GP PUK	0.57	0.79	0.56	0.77
GP Radial	0.65	0.69	0.65	0.68
PLS	0.29	0.97	0.30	1.00
SVM Norm. Polynomial (NP)	0.70	0.64	0.73	0.60
SVM Polynomial	0.71	0.63	0.71	0.62
SVM Radial	0.68	0.65	0.70	0.64
Family QSAR	0.31	0.70	0.31	0.96
		**Aminergic GPCRs Dataset**		
	$R_{\text{int}}^{2}$	**RMSEP**_**int**_	$R_{0\;\text{ext}}^{2}$	**RMSEP**_**ext**_
GP Bessel	0.56	0.83	0.56	0.80
GP Laplacian	0.62	0.78	0.63	0.75
GP Norm. Polynomial (NP)	0.69	0.68	0.72	0.66
GP Polynomial	0.68	0.71	0.70	0.68
GP PUK	0.46	0.93	0.46	0.90
GP Radial	0.69	0.69	0.71	0.66
PLS	0.69	0.69	0.27	1.05
SVM Norm. Polynomial (NP)	0.69	0.68	0.72	0.66
SVM Polynomial	0.69	0.69	0.71	0.66
SVM Radial	0.69	0.69	0.72	0.66
Family QSAR	0.38	0.98	0.38	0.97
		**Dengue virus NS3 proteases Dataset**		
	$R_{\text{int}}^{2}$	**RMSEP**_**int**_	$R_{0\;\text{ext}}^{2}$	**RMSEP**_**ext**_
GP Bessel	0.91	0.43	0.92	0.44
GP Laplacian	0.88	0.54	0.91	0.50
GP Linear	0.91	0.45	0.91	0.48
GP Norm. Polynomial (NP)	0.88	0.50	0.91	0.48
GP Polynomial	0.91	0.42	0.92	0.44
GP PUK	0.77	1.10	0.81	1.13
GP Radial	0.91	0.45	0.91	0.45
PLS	0.90	0.45	0.91	0.49
SVM Norm. Polynomial (NP)	0.86	0.54	0.91	0.46
SVM Polynomial	0.89	0.46	0.90	0.51
SVM Radial	0.90	0.48	0.90	0.48
Family QSAR	0.29	1.19	0.48	1.13

For the three datasets, the best models are obtained with GP, being the lowest RMSEP_ext_ and highest *R*^2^_0 *ext*_ values: (i) adenosine receptors: 0.58 and 0.75 with NP kernel, (ii) GPCRs: 0.66 and 0.72 with NP kernel, and (iii) Dengue virus NS3 proteases 0.44 and 0.92 with Bessel kernel. Overall, GP models for the three datasets agree with the validation criteria.

*Abbreviations*: *RMSEP* root mean square error in prediction, *Ext.* external, *Norm* Normalized.

#### GP statistics are within the limits of the theoretical maximum model performance

The distributions of maximum $R_{\text{ext}}^{2}$, $R_{0\;\text{ext}}^{2}$, and $Q_{\text{ext}}^{2}$ and minimum RMSEP_ext_ theoretical values, obtained as described in subsection Assessment of maximum model performance in Methods, are given in Additional file 1: Figure S2 for the three datasets. The mean value of the distribution of maximum $R_{0\;\text{ext}}^{2}$ values are equal to 0.80, 0.68 and 0.96 for the adenosine, GPCRs, and dengue virus NS3 proteases datasets, which highlights that the maximum correlation values that can be obtained when modeling public data are far from the optimal maximum correlation value of one. This is not surprising given the noise levels in public bioactivity data [22,23]. The best RMSEP_ext_ and $R_{0\;\text{ext}}^{2}$ values (Table 2) obtained with GP are respectively: 0.58 and 0.75 (adenosine receptors), 0.66 and 0.72 (GPCRs), and 0.44 and 0.92 (dengue virus NS3 proteases), which remain in the limits of these extreme theoretical values (Additional file 1: Figure S2), thus supporting the suitability of our modeling pipeline to handle data uncertainty. The mean values of the theoretical RMSEP distribution were close to the experimental uncertainty on bioactivity, for the adenosine receptors and the dengue virus NS3 proteases datasets, with respective mean RMSEP_ext_ values of 0.54 *pK*_*i*_ units and 0.27 log units (Additional file 1: Figure S2). However, the mean RMSEP_ext_ value increases up to 0.68 *pK*_*i*_ units for the GPCRs dataset owing to its larger size and sparsity.

#### PCM outperforms QSAR on the studied datasets

A comparison between models trained on only compound descriptors (‘Family QSAR’) [62] and PCM permits to assess whether the use of GP improved the bioactivity modeling, by simultaneously modeling the target and the chemical spaces within a PCM study [6]. Indeed, radial kerneled Family QSAR models with ligand descriptors (Table 2) failed to model the data, being the RMSEP_ext_ and $R_{0\;\text{ext}}^{2}$ values respectively: 0.96 and 0.31 (adenosine receptors), 0.97 and 0.38 (GPCRs), and 1.13 and 0.48 (dengue virus NS3 proteases).

#### Strong mapping power of the normalized polynomial kernel

Radial and polynomial kernels have been traditionally used in QSAR and PCM modeling, [16,63] but the versatility of other kernels for bioactivity modeling has been recently demonstrated [63-65]. To investigate this point in the frame of GP models, we compared the performance of various kernels (Bessel, Laplacian, NP, and PUK) with the radial and polynomial kernels.

As described above, in contrast to Huang *et al.*, [63] we found the normalized polynomial (NP) kernel to have enough mapping power to model the three datasets (Table 2). Nonetheless, in the case of the dengue virus NS3 proteases dataset, although NP kernel produces a statistically correct modeling with RMSEP_ext_ and $R_{0\;\text{ext}}^{2}$ values of 0.48 and 0.91, it is slightly outperformed by the Bessel kernel, which displays respective RMSEP_ext_ and $R_{0\;\text{ext}}^{2}$ values of 0.44 and 0.92 (Table 2). The PUK kernel [65] exhibited strong mapping power in previous studies of HIV-1 proteases and histone deacetylases (HDAC) inhibitors, [63,64] but in the present study we could not obtain satisfactory models for none of the three datasets. The Laplacian and Bessel kernels allow a proper mapping of the three datasets with $R_{0\;\text{ext}}^{2}$ values within the range 0.60–0.90 (see Table 2 for further details).

For the adenosine receptors dataset, different statistics values are observed between the internal and external validation, as the RMSEP_ext_ values are larger for the radial kernel (0.68) than for the polynomial and Bessel kernels (0.63 in both cases). Nonetheless, a different picture is observed for RMSEP_int_, as the values for the radial, polynomial and Bessel kernels are 0.69, 0.64 and 0.70 *pK*_*i*_ units. Although RMSEP_ext_ and RMSEP_int_ values are similar, the small increase of RMSEP_ext_ with the Bessel kernel might suggest a slight degree of overfitting [66].

#### GP and SVM perform *on par*

**Figure 1:**
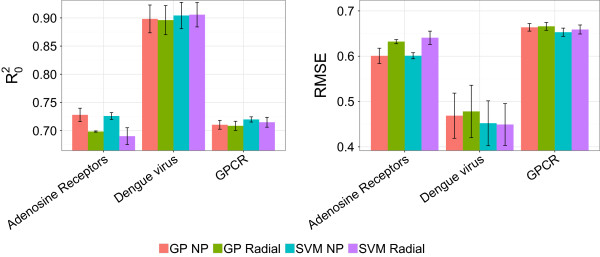
**Comparison between the performance of GP and SVM with either the radial or the normalized polynomial (NP) kernel.** Ten models were calculated for each dataset and for each combination of modeling technique and kernel, thus resulting in a total of 60 models. The performance of GP and SVM was assessed by kernel for the three datasets. Given that the distributions of RMSEP_ext_ and $R_{0\;\text{ext}}^{2}$ values were normally distributed, a two-tailed *t*-test of independent samples was applied to statistically evaluate their differences. These analyses let us conclude that SVM and GP perform *on par* for the modeling of the three datasets considered in this study.

The performance of the GP and SVM models was compared for each dataset using the radial, the polynomial, and the NP kernels, as the first two are the most widespread kernels within the modeling community [15,16,63]. Using different seed values, we trained ten different models for each modeling technique and dataset, resulting in a total of 60 models (Figure 1). To be able to statistically test the difference between the models results, distributions of the RMSEP _*e* *x* *t*_ and $R_{0\;\text{ext}}^{2}$ were generated for each kernel/dataset combination. Both RMSEP_ext_ and $R_{0\;\text{ext}}^{2}$ statistics were normally distributed in all cases (Shapiro-Wilk normality test, *α* 0.05), and a two-tailed *t*-test of independent samples (*α* 0.05) was applied to compare the behavior of SVM and GP. As it can be seen in Figure 1 and from the result of the *t*-test, both SVM and GP perform on *par* in the three case studies for radial and NP kernels. Similar results (data not shown) were obtained for the polynomial kernel.

To probe the linearity of the datasets, we trained linear PLS models. For two datasets, PLS appears unable to infer the complex (non-linear) relationships within the data, leading to RMSEP_ext_ and $R_{0\;\text{ext}}^{2}$ of 1.00 and 0.30 for the adenosine receptors, and 1.05 and 0.27 for the GPCRs datasets, respectively (Table 2). At contrary, the dengue NS3 proteases dataset presents a clearly linear relationship, with RMSEP_ext_ and $R_{0\;\text{ext}}^{2}$ values of the PLS model of 0.49 and 0.91. However, on the same dataset, the model obtained with a linear kerneled GP model slightly outperformed PLS, with respective RMSEP_ext_ and $R_{0\;\text{ext}}^{2}$ values of 0.48 and 0.91.

#### Noise influence on GP depends on the kernel

**Figure 2:**
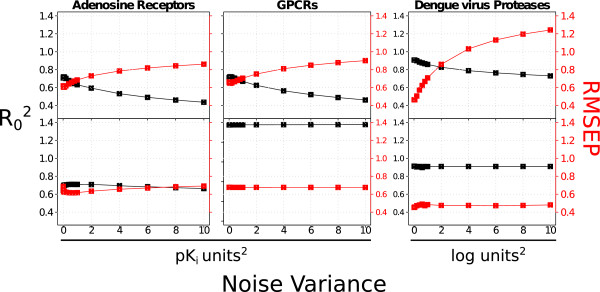
**Noise influence in model performance.** RMSEP_ext_ (red) and $R_{0}^{2}\;\text{ext}$ (black) values obtained when increasing the noise level (noise variance added to the diagonal of the covariance matrix) were calculated for: adenosine receptors (left figure), GPCRs (medium figure) and dengue virus NS3 proteases (right figure). Upper plots correspond to GP models calculated with the radial kernel while the bottom plots refer to GP models with the normalized polynomial (NP) kernel. In all cases, the radial kernel appears more sensitive to noise, while the NP kernel performs equally well when noise is added to the data. These data suggest that the NP kernel is more appropriate for the modeling of noisy PCM datasets.

RMSEP _*e* *x* *t*_ and $R_{0\;\text{ext}}^{2}$ were calculated for adenosine receptors, GPCRs, and dengue virus NS3 proteases for different levels of noise $\sigma_{d}^{2}$ added to the diagonal of the covariance matrix *C*_*X*_ (Equation 2). The results obtained for radial kernels (Figure 2, upper plots) appear more sensitive to the noise than the ones obtained for NP kernels (Figure 2, bottom plots), for which the variations of the RMSEP_ext_ and $R_{0\;\text{ext}}^{2}$ sets are lower than 0.10 *pK*_*i*_ or log units. This trend is more obvious for the dengue virus NS3 proteases dataset, probably originating from the small size of this dataset. The polynomial kernel (data not shown) displays robustness similar to those of NP kernel. These analyses suggest that NP or polynomial kernels would constitute a reasonable choice when modeling noisy data. To summarize, GP models perform *on par* with SVM and outperform Family QSAR and PLS on the three datasets. The NP kernel leads to the best GP models being also the most tolerant kernel to noisy bioactivities. GP models trained on the dengue virus NS3 proteases systematically display better metrics than the other datasets, likely due to the high matrix completeness (88.84%) of this dataset (Table 1).

### Predicted confidence intervals follow the cumulative density function of the Gaussian distribution

#### GP predictions mostly follow the cumulative Gaussian distribution

**Figure 3:**
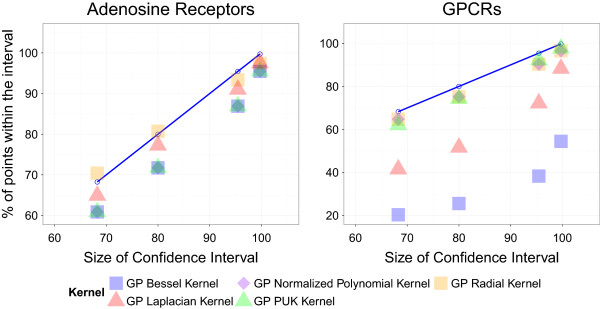
**Analysis of the confidence intervals predicted on (left) the adenosine receptors and (right) aminergic GPCRs external sets.** The percentages of annotated values lying within the intervals of confidence of 68%, 80%, 95% and 99% (ordinate axis) are depicted versus the size of the intervals. The blue line defines the theoretical proportionality between the size of confidence intervals and the number of points within the intervals, in the frame of the Gaussian cumulative function. The radial, PUK, and normalized polynomial (NP) kernels are in close conformity with the cumulative Gaussian distribution in both datasets, while the Laplacian and Bessel exhibit a diverse behavior depending on the dataset. Therefore, GP provide prediction errors in agreement with the Cumulative Gaussian distribution which can be reliably used to define intervals of confidence for the predictions.

To analyze the reliability of the error bars obtained with GP with the tested kernels, different intervals of confidence (IC) for each predicted bioactivity value on the external set were defined, namely: 68%, 80%, 95%, and 99%. Subsequently, the percentage of compound-target combinations for which the experimental bioactivity value lied within the bounds of each interval was calculated. Following the cumulative density function of the Gaussian distribution (cumulative Gaussian distribution), [33] the percentage of satisfactory cases should be proportional to the interval size.To test this hypothesis, the percentages of predicted bioactivities for which the experimental values were within the confidence intervals were compared to the size of these intervals (Figure 3). As the small size of the dengue virus NS3 proteases did not allow a good sampling of the Gaussian distribution, this dataset was not included in the comparison. This analysis was thus performed for the adenosine receptors and GPRCs datasets with the Bessel, Laplacian, NP, PUK, and radial kernels. It is noteworthy that the predicted variance obtained with the polynomial kernel is much larger than the range of bioactivity values, thus making impossible to evaluate their concordance with the cumulative distribution. However, the NP kernel allows to obtain values within the interval {0,1} for the predicted variance thanks to its normalized formulation.The experimental values for the radial kernel match the theoretically expected behavior, represented on Figure 3 by bullet points connected by a blue line, and calculated in the context of a Gaussian cumulative function. The match between experiment and theory holds for the PUK and NP kernels for both datasets. The difference between the cumulative Gaussian distribution and the different intervals of confidence calculated for the Adenosine receptors dataset is around 10% for the other kernels (Figure 3, left plot). By contrast the Bessel and Laplacian kernels do not provide informative intervals of confidence for the GPCRs dataset (Figure 3, right plot).

#### GP determine the applicability domain of the model

**Figure 4:**
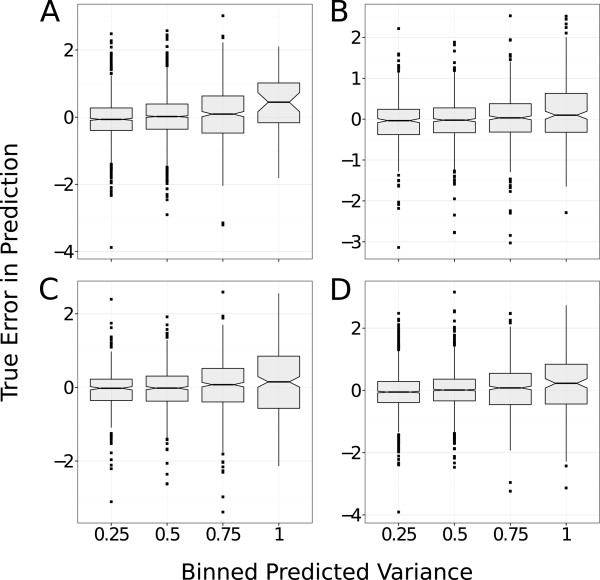
**GP determine models applicability domain.** The differences between the true and predicted bioactivities (y axis) and the errors on predictions estimated by the GP model (x axis) are compared for the adenosine receptor dataset with radial **(A)** and NP **(B)** kernel, and for the GPCRs dataset with radial **(C)** and NP **(D)** kernels. The distribution of the differences between true and predicted bioactivities increases with the GP error on the prediction. This validates that the GP error is a measurement of the Applicability Domain (AD) of the model.

The variance predicted with GP models, $\sigma_{y^{⋆}}^{2}$, quantifies how much information the model can infer from the data (Eq. 5). Therefore, we hypothesized that: the distribution of the differences between the predicted and the observed bioactivity values, are more dispersed for compound-target pairs distant from the training set (high values of $\sigma_{y^{⋆}}^{2}$). To verify this hypothesis, we binned the external set into four groups depending on the value of the predicted variance: {0.25,0.5,0.75,1}. The differences between true and predicted bioactivities were compared (Figure 4) to the bioactivity errors predicted in the GP model. This analysis was done on the adenosine receptors and GPCR datasets for the predicted variances obtained with the NP and the radial kernels. As the dispersion of the distribution of the differences increases with the errors predicted by GP, irrespective of the kernel or dataset considered, this error can be thus considered as a reliable estimate of the applicability domain (AD).

Interestingly, while the average differences between predicted and observed bioactivities are close to zero for the subsets of GP errors of 0.25, 0.5 and 0.75, this average value is biased towards few tenths of a *pK*_*i*_ unit (Figure 4) for the subset displaying the largest GP error. This observation indicates that errors on bioactivities are underestimated by the GP model for compound-target pairs distant from the training set. GP models with the NP and radial kernels provide prediction errors in agreement with the cumulative Gaussian distribution, which is the maximum theoretical precision attainable. Furthermore, the applicability domain of GP models can be determined from the errors predicted by GP.

### Analysis of GP performance *per* target

To further understand the predictive capability of GP models on each analyzed target, we trained ten GP models with the NP kernel. Different seed values were used for the generation of the training and the external sets. Once the GP predictions have been obtained, we divided the external set into subsets grouped by target, and calculated average $R_{0\;\text{ext}}^{2}$ and RMSEP_ext_ values on these subsets. This analysis per target was conducted only on the datasets of adenosine receptors and GPCRs, because of their large sizes and numbers of involved targets.

#### Adenosine receptors

**Figure 5:**
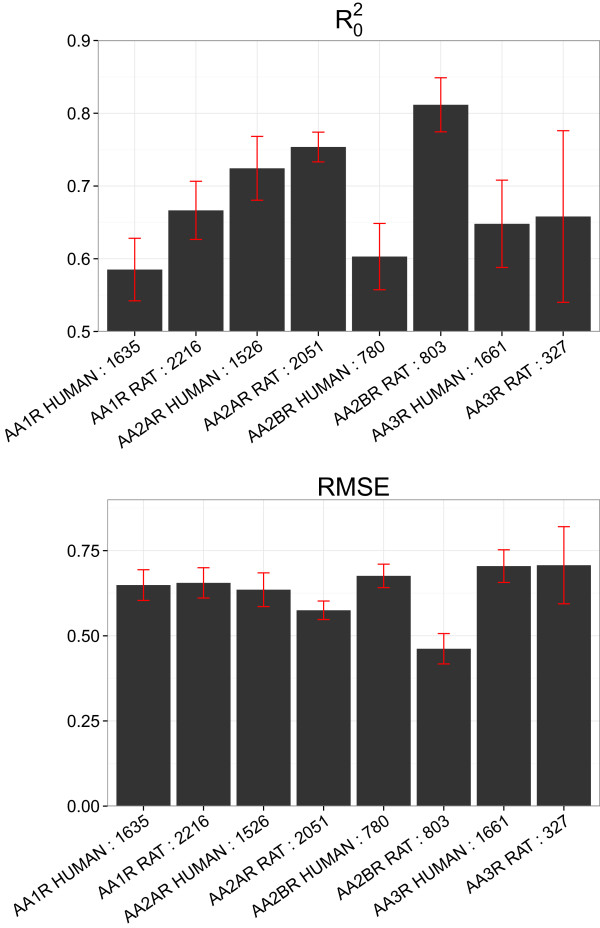
**Model performance per target on the external set for the adenosine receptors dataset.** The upper panel corresponds to $R_{0\;\text{ext}}^{2}$, while the lower panel to RMSEP_ext_. These values were averaged for ten models trained on each subset corresponding to a given target. The best modeled target is the rat adenosine *A*_2*b*_ receptor (AA2BR RAT), while the worst is the rat *A*_3_ receptor (AA3R RAT). In all cases, the mean RMSEP_ext_ values are below 0.75 *pK*_*i*_ units, indicating that GP modeling can predict compounds bioactivities on subsets corresponding to a given target.

The highest mean RMSEP_ext_ value is between 0.70 and 0.75 *pK*_*i*_ units, and the lowest mean $R_{0\;\text{ext}}^{2}$ value is 0.62 (Figure 5). In this dataset, the performance is not directly related to the number of compounds annotated *per* target. Indeed, the best result is obtained on the rat *A*_2*b*_ receptor (AA2BR RAT, 803 compounds) whereas one of the worst results is displayed by the human *A*_1_ receptor (AA1R HUMAN, 1635 compounds).

On the other hand, the results cannot be related to the chemical diversity of the compounds, analyzed with pairwise Tanimoto similarity (Additional file 1: Figure S3). Indeed, the two targets displaying the largest variability in the range of 0.50–0.75 Tanimoto similarity are rat *A*_3_ (AA3R RAT) and human *A*_2*b*_ (AA2BR HUMAN), for which quite different performances are observed (RMSEP_ext_ in the 0.70–0.75 range and in the 0.59–0.61 range respectively: Figure 5). Similarly, human *A*_1_ (AA1R HUMAN) and *A*_2*a*_ (AA2AR HUMAN) receptors, display the smallest variability for compounds, and show quite different levels of performance ($R_{0\;\text{ext}}^{2}$ in the 0.56–0.60 range and in the 0.70–0.74 range respectively).

The lack of connection between the performance and the chemical diversity could arise from the binding site residue selection, which might not be equally suited for all adenosine receptors. This is supported by two other facts, namely: (i) the differences in extracellular loop length that are known for the adenosine receptor paralogues; and (ii) secondly the knowledge that these loops are important for ligand binding [67-69].

#### GPCRs

In the GPCR dataset, the best RMSEP_ext_ (Additional file 1: Figure S4) and $R_{0\;\text{ext}}^{2}$ (Additional file 1: Figure S5) values are obtained on target subsets with a number of annotated compounds larger than 200 (in grey in Additional file 1: Figures S4 and S5). Between the subsets, no major differences in performance are observed for an amount of annotated compounds between several hundreds and over 1500. It is however noticeable that the predictive ability of the models increased as the target space included in the training dataset broadened. Indeed, a bioactivity selection previously done including information from 26 human aminergic GPCRs (4,951 datapoints), marked with an asterisk in Additional file 1: Table S2, did not produce any sound statistical metrics, as $R_{0\;\text{ext}}^{2}$ values lower than 0.40 were obtained whatever the kernel or machine learning algorithm used. But, the addition to the first selection of the bioactivities measured on mammal orthologues improved the prediction, although some of the additional bioactivity sets were singletons (Additional file 1: Table S2).

A large diversity of performance with RMSEP_ext_ values in the range of 0.00–2.50 *pK*_*i*_ units is observed for the targets annotated with one compound (Additional file 1: Figure S4). A relationship can be nevertheless established between these performances and the number of annotated compounds on orthologues proteins. For example, the 5-HT2C mouse receptor (5HT2C MOUSE) annotated with three compounds exhibits a mean RMSEP_ext_ value between 0.00 and 0.20 *pK*_*i*_ units (Additional file 1: Figure S4), because 345 and 558 compounds are respectively annotated on the orthologue rat and human 5-HT2C receptors. The good performance obtained for this mouse receptor is probably due to the similarity of the 345 and 558 compounds to the ones annotated to the 5-HT2C mouse receptor. The importance of various targets for GP prediction was assessed for the adenosine receptors and GPCRs datasets. To obtain statistically validated models, a balance has to be found between two trends: (i) the inclusion of bioactivity information from orthologues improves the predictive ability of the models for both datasets, but (ii) an increase of the chemical diversity might hamper the acquisition of sound models as shown for the adenosine receptors dataset.

### Model interpretation of ligand descriptors

#### Compounds bioactivity depends on multiple weak contributions of chemical substructures

The influence of the substructures on compound bioactivities, for both the adenosine receptors and the GPCRs, was analyzed as described in section Interpretation of ligand substructures. In the present study, the contribution of more than 90% of substructures to the *pK*_*i*_ values is close to zero (black regions in Additional file 1: Figure S6). We observed similarly (data not shown) that chemical substructures contributing in a very variable way to the *pK*_*i*_ values (average contribution equal to zero and standard deviations in the range of 0.50 - 1.00 *pK*_*i*_ units), are present in sets of compounds displaying large variability in experimental bioactivity on a given target.

Hence, more than 90% of the substructures from the datasets analyzed here, display alternatively the following properties: (i) they are not implicated in compound bioactivity as their presence or absence does not influence compounds bioactivity, (ii) their contribution to the *pK*_*i*_ values, is conditioned to the presence or absence of other substructures [70].

The highest contributions to the *pK*_*i*_ values, on both the GPCRs and the adenosine receptors datasets, is close to 1 *pK*_*i*_ units (Additional file 1: Figure S6), in the range similar to those obtained by van Westen *et al.*[15]. Therefore, even those few substructures with a large contribution, highlighted in Additional file 1: Figure S6, do not explain a large proportion of the bioactivity.

#### ARD provides a biologically meaningful interpretation of PCM models

**Figure 6:**
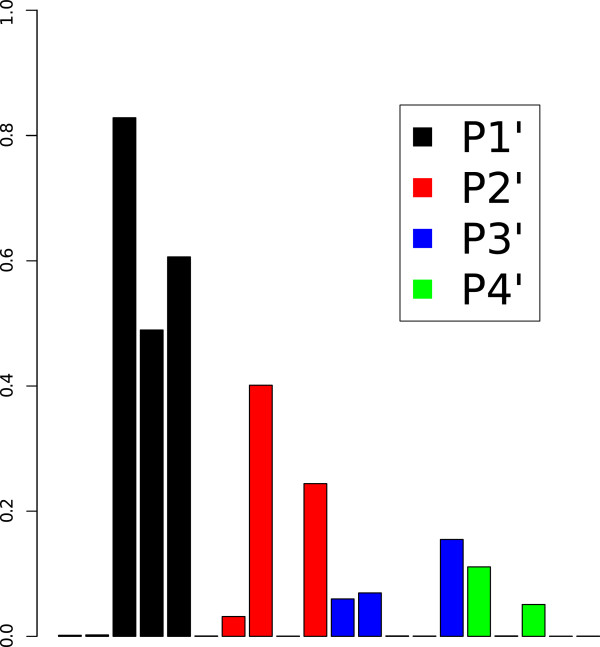
**Descriptor importance for the dengue virus NS3 proteases dataset.** Descriptor importance is calculated in the frame of Bayesian Automatic Relevance Determination (ARD) as the inverse of the value of the length scale of each descriptor. The descriptors of the first and second residues of the tetra-peptides (positions P1’ and P2’) are the most relevant for the model. This is in agreement with the higher influence of these two substrate positions for the cleavage rates of the proteases.

The substrates in the dengue virus NS3 proteases dataset are tetra-peptides. The relative importance of the four residues of these tetra-peptides was deconvoluted in the frame of ARD, described in Materials and Methods, by taking the inverse of the optimized *l* value of the radial kernel (Figure 6). The largest inverse values are obtained for P1’ followed by P2’, P3’ and P4’ displaying similar values. Thus, the first amino acid (P1’) is the most relevant for the model followed by the second one (P2’), in contrast to the third and fourth ones (P3’ and P4’). In the study of Prusis *et al.*, [41] the PLS coefficients with the highest values correspond to the first and second amino acids, as it is also the case here. A further detailed comparison of the PLS and the presented GP model is beyond the scope of this study. However, it should be noticed that the descriptors used in the present study and in Ref [41]. differ: 5 z-scales in our case versus 3 z-scales, C7.4, t1-Rig, and t2-Flex [71] in the PLS model. Although the PLS and GP models might assign different weights to the different descriptors, they both identify the first amino acid position as having the largest influence on *K*_*c* *a* *t*_, in agreement with experimental results [41].

GP models were interpreted on the basis of ligand descriptors. For datasets where ligands are compound descriptors (GPCRs and adenosine receptors datasets), the interpretation was not conclusive. By contrast, the interpretation of GP models according to the amino acids of the tetra-peptide ligands in the dengue datasets gave biologically meaningful results, in agreement with the scientific literature [41]. In that way, ARD can be applied to biologically interpret systems: *e.g.* identify residues responsible for compound binding. Additionally, ARD with the radial kernel can model non-linear relationships, which is not possible with PLS without the introduction of (not easily interpretable) cross-terms [6,41].

## Discussion

In the present study, we have demonstrated that Gaussian Processes (GP) allow to predict compound bioactivities on biomolecular targets. The statistically soundness of GP models is observed for a broad panel of kernels, among which the NP and radial kernels display the best results. GP and SVM display statistically similar performance for the modeling of multispecies proteochemometric datasets of different sizes. Moreover, Family QSAR and PCM models were trained on the same number of datapoints and PCM produced much better results than Family QSAR, due to the introduction of target descriptors.

GP were applied on the following datasets: two large datasets involving GPCRs and adenosine receptors and one small dataset (199 datapoints) comprising four dengue NS3 proteases. The dengue dataset exhibits a high degree of linearity, as demonstrated by the high performance of both PLS and GP with a linear kernel on this dataset. Unsurprisingly, a better performance of GP is observed with different kernels for the dengue dataset than for the two other ones, due to the high matrix completeness in the dengue dataset and to its linearity. The satisfactory results obtained for the dengue dataset encourages the application of GP to model relatively small datasets issued from a single laboratory. The use of such in-house datasets would reduce the bias introduced by annotation errors and by the use of non-normalized experimental conditions.

The inclusion of chemical and target information from several organisms (orthologues) increases model performance and the applicability of models to predict bioactivity for new compound target-combinations. These observations are in favor for the routine inclusion of multispecies bioactivity information in PCM settings. These results disagree with Gao *et al.*[72], who stated that the addition of orthologues to human aminergic GPCRs would reduce the AD. Our understanding of the results obtained here is that the incorporation of bioactivity data from a wide range of species led to a significant increase of models performance given that binding patterns tend to be conserved among orthologues [73]. We have seen on the GPCR dataset, that the inclusion of singletons compounds bioactivities on human orthologues helps to increase models performance. This may be of tremendous relevance in the often encountered cases where limited bioactivity information is known on a given human target, but a much larger number of bioactivities have been measured on orthologues of this target [16,73,74]. Our results suggest that the chemical diversity considered and the number of datapoints have to be balanced to obtain sound models while exhibiting proper predictive abilities.

An additional outcome of GP with respect to SVM is the estimation of the uncertainty of predictions. Indeed, the Bayesian formulation of GP permits to obtain intervals of confidence for individual predictions defined from the GP predicted variance. These intervals were shown to be in agreement with the cumulative Gaussian distribution when using the radial and NP kernels, but not always for the Bessel or Laplacian kernels, highlighting that the kernel choice has to be made in the light of both models performance and reliability of the predicted variances. We have also shown here that GP using as covariance function the polynomial or the NP kernel can handle noisy datasets, as the GP performance is only slightly affected when noise is introduced in the data. Nonetheless, each kernel should be chosen in the light of underlying structure of the dataset, as the kernel controls the prior distribution over functions, and thus the models generalization properties [48,75]. It is noteworthy to mention that we have implemented a broad, though not exhaustive, panel of kernels, which is susceptible to be further completed with other base kernels or kernel combinations (composite kernels) [48,75,76].

GP can consider individual experimental errors as input for the probabilistic model which may constitute a preeminent advantage when gathering information from diverse sources, each of which including distinct levels of experimental uncertainty [33]. In the present study, an approximation of the experimental uncertainty of heterogeneous *pK*_*i*_ values, recently reported by Kramer *et al.*[22] to exhibit a standard deviation of 0.54 *pK*_*i*_ units, has been introduced in the model. Nonetheless, GP allow the inclusion of the uncertainty of each individual datapoint into the model, which might lead to a more accurate modeling pipeline in cases where the experimental uncertainty of each datapoint is available.

Traditionally, the application of GP to model large datasets has been limited since the inversion of the covariance matrix scales with the cube of its dimension, *i.e.* GP is an algorithm of complexity *O*(*N*^3^) [31,48]. In the present study, we have not reported training times since models have been trained with GP implementations coded in different programming languages (subsection Machine learning analyses and implementation). In the experience of the authors, the application of ARD is limited by the size of the datasets, being not applicable in practice to datasets with more than several thousands of datapoints, or with more than several hundreds of descriptors. Nevertheless, new GP implementations have proved to seemingly decrease calculation times, [77-79] which might increase the applicability of GP to large PCM datasets in the future.

Overall, we have shown here that GP simultaneously provides bioactivity predictions and assessment of their reliability. The application of GP to PCM datasets, gives the insight that GP could also be very useful in the drug discovery for personalized medicine, when the target space includes several mutants of a given target [15,80]. In the same way, GP could even be used in the context of decision making in clinics [81].

## Conclusion

Gaussian Processes (GP) have been proposed and tested for the prediction of bioactivity measurements, and found to perform at the same level of statistical significance as Support Vector Machines (SVM). In addition, GP is the only method, up to now, to give predictions as probability distributions, thus permitting the estimation of errors on the bioactivity predictions as well as an estimation of the applicability domain. Moreover, GP are tolerant to noisy bioactivities. GP models trained on PCM datasets can also be used to analyze the effect of ligand features (compound substructures or peptide residues).

## Competing interests

The authors declare that they have no competing interests.

## Authors’ contributions

ICC, AB, and TM designed the study. ICC trained the models, analyzed the results and prepared the figures. GvW, EBL, and ICC provided datasets and descriptor calculations. DM provided analytical tools. ICC, GvW, AB, and TM wrote the paper. All authors read and approved the final manuscript.

## Supplementary Material

Additional file 1 **Supplementary information.** This file contains (i) the modeling pipeline used in this study, (ii) supplementary figures, and (iii) supplementary tables. Click here for file
