# Supplementary material for: Proteochemometric modeling in a Bayesian framework
Source: J Cheminform. 2014 Jun 28;6:35. doi: 10.1186/1758-2946-6-35 (PMC4083135; doi:10.1186/1758-2946-6-35)
Supplement: Additional file 1 — Supplementary information. This file contains (i) the modeling pipeline used in this study, (ii) supplementary figures, and (iii) supplementary tables. [file 1758-2946-6-35-S1.pdf]

Supplementary Information: Proteochemometric Modeling in a Bayesian Framework

Isidro Cortes-Ciriano[1], Gerard J.P. van Westen[2], Eelke B. Lenselink[3], Daniel S. Murrell[4], Andreas Bender[4*], Thérèse E. Malliavin[1*]

(1) Institut Pasteur, Unité de Bioinformatique Structurale; CNRS UMR 3825; Département de Biologie Structurale et Chimie; 25, rue du Dr Roux, 75015 Paris, France.
(2) European Molecular Biology Laboratory. European Bioinformatics Institute Wellcome Trust Genome Campus, Hinxton, United Kingdom.
(3) Division of Medicinal Chemistry, Leiden Academic Center for Drug Research, Leiden, The Netherlands.
(4) Unilever Centre for Molecular Science Informatics, Department of Chemistry, University of Cambridge, Cambridge, United Kingdom.

Corresponding author : Thérèse E. Malliavin; E-mail: terez@pasteur.fr; Phone: +33 1 45 68 88 54
Andreas Bender; E-mail: ab454@cam.ac.uk; Phone: +44 (1223) 762 983

Keywords : Proteochemometrics, Bayesian Inference, Gaussian Process, Chemogenomics, GPCRs, Applicability Domain.

*To whom correspondence should be addressed

Supplementary Materials and Methods

Data Preprocessing and *In Silico* Modeling Pipeline

Descriptors with variances close to zero, were detected by computing for every descriptor the ratio between the frequency of occurrences of the two most represented values. If this ratio is larger than 30, the descriptor variance is considered as negligible, and the descriptor removed. Afterward, the descriptors are centered to zero mean and scaled to unit variance prior to any modeling. In the case of the Dengue virus NS3 proteases dataset, k_{cat} values were logarithmically transformed as done by Prusis *et al.*[1] In addition, each descriptor type was scaled as $1/(\sqrt{k})$, being k the number of descriptors of a given type (target, substrate or cross-term).

Model Training and Validation

The predictive ability of the generated models was assessed by k-fold cross validation (CV).[2] The whole dataset was split into $k+1$ folds by stratified sampling of the bioactivity values. One fold ($1/k+1$) constitutes the external (hold-out) set, while the remaining ($k/k+1$) are used to train models. To significantly compare the quality of the modeling with different kernels or machine learning approaches, the same folds were created on each dataset. Both model performance and over-fitting were assessed by monitoring their predictive ability on the external set.[2]

Models are built on the training set in the following way: at each step, one fold is removed from the training set, ($k/k+1$), and a model is trained for each combination

of hyperparameters. Subsequently, the bioactivity of the removed fold is predicted with each of these models, and the performance of the model assessed by the coefficient of correlation, R^2, and the root mean square error in prediction (RMSEP)[3] between the predicted and the observed values. This step is repeated k times, each time holding out a different fold. Subsequently, the average values of R^2 and RMSEP over the k models is calculated for each combination of hyperparameters. The combination of hyperparameters exhibiting the lowest average RMSEP and the highest average R^2 provides the internal validation: $\text{RMSEP}_{\text{int}}$ and R^2_{int} (also known as q^2 or CV R^2) (Equations (S1) and (S2)). These values are used to train the final model on the whole training set ($k/k+1$). To assess both model predictive ability and performance, this model is then used to predict the bioactivity of the external set ($1/k+1$) which provides the external validation by calculating $\text{RMSEP}_{\text{ext}}$ (Equation S3) and other correlation metrics (Equations (S4) to (S6)). For the adenosine receptors and aminergic GPCRs k was taken equal to 5 and for the Dengue virus proteases equal to 10.[1]

Both internal and external validation were performed according to the criteria proposed by Tropsha *et al.*[4–6] (Equations (S7) to (S10)), and to the $\text{RMSEP}_{\text{int}}$ (Equation S1) and $\text{RMSEP}_{\text{ext}}$ (Equation S3) metrics.

Internal validation

The statistical metrics R^2_{int} and $\text{RMSEP}_{\text{int}}$ are defined as:

$$RMSEP_{int} = \frac{\sqrt{(y_i - \widetilde{y}_i)^2}}{N} \tag{S1}$$

$$R^2_{int} = 1 - \frac{\sum_{i=1}^{N}(y_i - \widetilde{y}_i)^2}{\sum_{i=1}^{N}(y_i - \bar{y}_{tr})^2} \tag{S2}$$

where N, y_i, $\widetilde{y}_i$ and $\bar{y}_{tr}$ represent respectively the size of the training set and the observed, the predicted and the averaged values of the response variable for those datapoints included in the training set. The data-set is indexed by i.

External validation

The statistical metrics $\mathrm{RMSEP_{ext}}$, Q^2_{ext}, R^2_{ext} and $R^2_{0\,ext}$ are defined as:

$$RMSEP_{ext} = \frac{\sqrt{(y_i - \widetilde{y}_i)^2}}{N} \tag{S3}$$

$$Q^2_{ext} = 1 - \frac{\sum_{j=1}^{N}(y_j - \widetilde{y}_j)^2}{\sum_{j=1}^{N}(y_j - \bar{y}_{ext})^2} \tag{S4}$$

$$R_{ext} = \frac{\sum_{i=1}^{N}(y_i - \bar{y}_{ext})(\widetilde{y}_i - \bar{\widetilde{y}}_{ext})}{\sqrt{\sum_{i=1}^{N}(y_i - \bar{y}_{ext})^2 \sum(\widetilde{y}_i - \bar{\widetilde{y}}_{ext})^2}} \tag{S5}$$

$$R^2_{0\,ext} = 1 - \frac{\sum_{j=1}^{N}(y_j - y_j^{r0})^2}{\sum_{j=1}^{N}(y_j - \bar{y}_{ext})^2} \tag{S6}$$

where N, y_j, $\widetilde{y}_j$ and $\bar{y}_{ext}$ represent respectively the size of the training set and the observed, the predicted and the averaged values of the response variable for those datapoints comprising the external set. An additional criterion is the value of the slope, s, which corresponds to the slope of the regression through the origin, $y_j^{r0} = s\widetilde{y}_j$. For a detailed discussion of both the evaluation of the predictive ability through the external set and different formulations for Q^2, see ref.[3] To be considered as predictive, a model must satisfy the following criteria:[4,6]

$$q_{int}^2 > 0.5 \tag{S7}$$

$$R_{ext}^2 > 0.6 \tag{S8}$$

$$\frac{(R_{ext}^2 - R_{0\,ext}^2)}{R_{ext}^2} < 0.1 \tag{S9}$$

$$0.85 \leq s \leq 1.15 \tag{S10}$$

Supplementary Figures

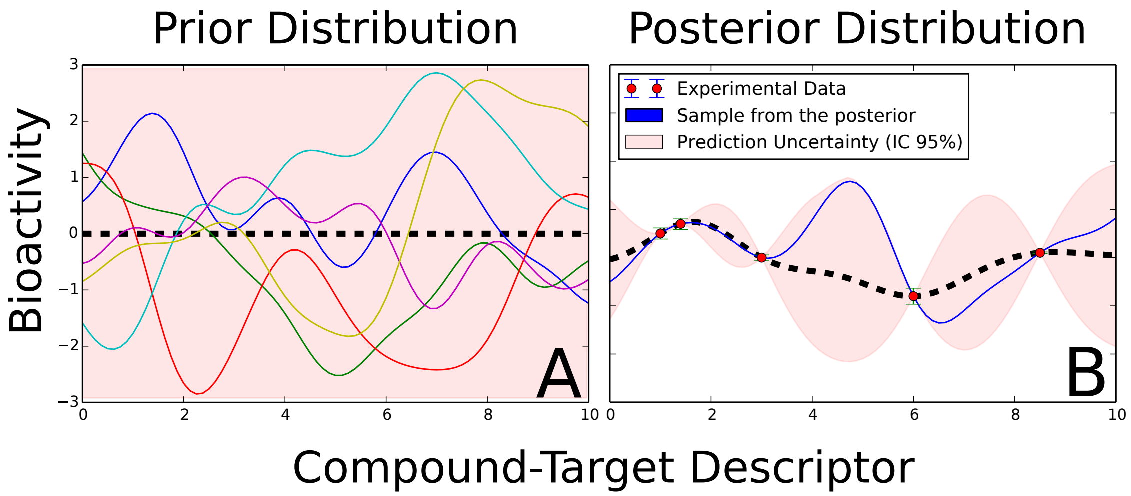

Supplementary Material, Figure S1: Illustrative example of GP theory in a two-dimensional problem. The prior probability distribution (A) embraces all possible functions which can potentially model the dataset. The mean of this distribution (black dotted line) is normally set to zero. In B, the inclusion of bioactivity information (red dots) accompanied by its experimental uncertainty (blue error bars) updates the prior distribution into the posterior probability distribution. Only those functions in agreement with the experimental data are kept. It is readily apparent that the uncertainty (red shadowed areas) notably increases in areas containing little experimental information. The average curve of the posterior distribution (black dotted curve) is considered as the best fit to the data.

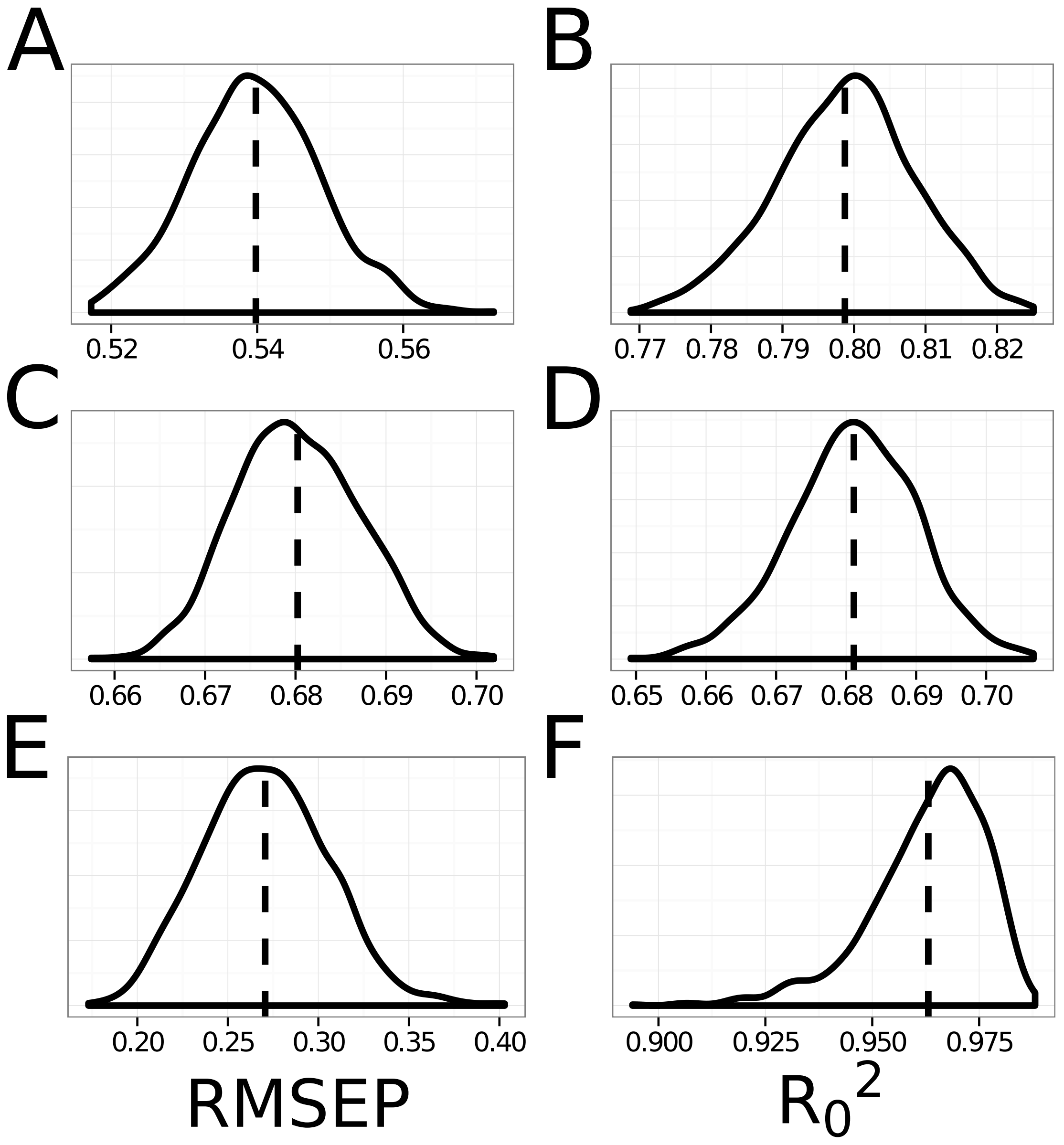

Supplementary Material, Figure S2: Distribution of the maximum theoretical values of $RMSEP_{ext}$ (A, C and E) and $R^2_{0\ ext}$ (B, D and F) for the adenosine receptors (A, B), GPCRs (C, D) and Dengue virus NS3 proteases datasets (E, F). These curves permit to estimate the reliability of $R^2_{0\ ext}$ and $RMSEP_{ext}$ obtained for the GP models.

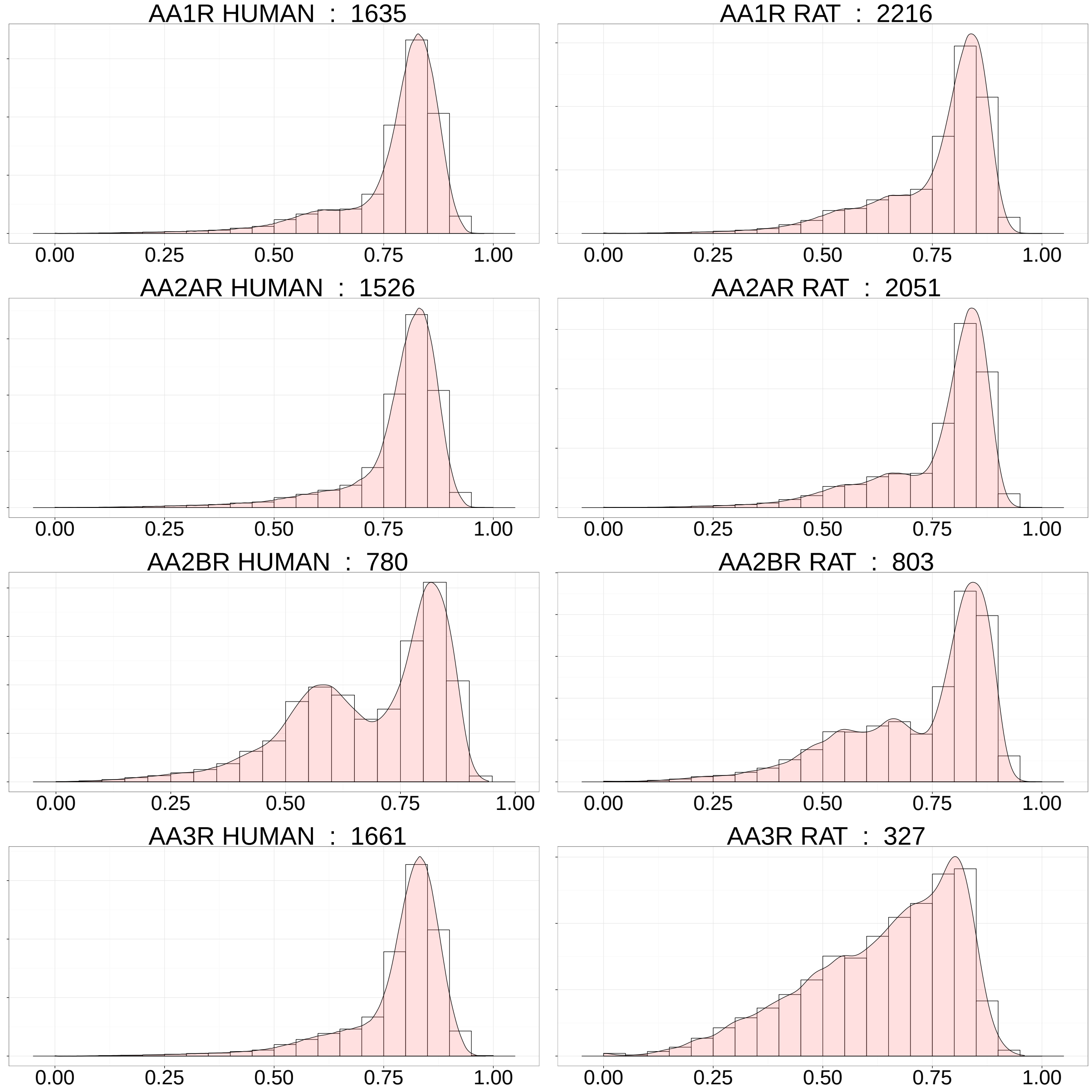

Supplementary Material, Figure S3: Distribution of pairwise compound Tanimoto similarity calculated on the target subsets extracted from the adenosine receptors dataset. The overall mean pairwise similarity is around 0.8.

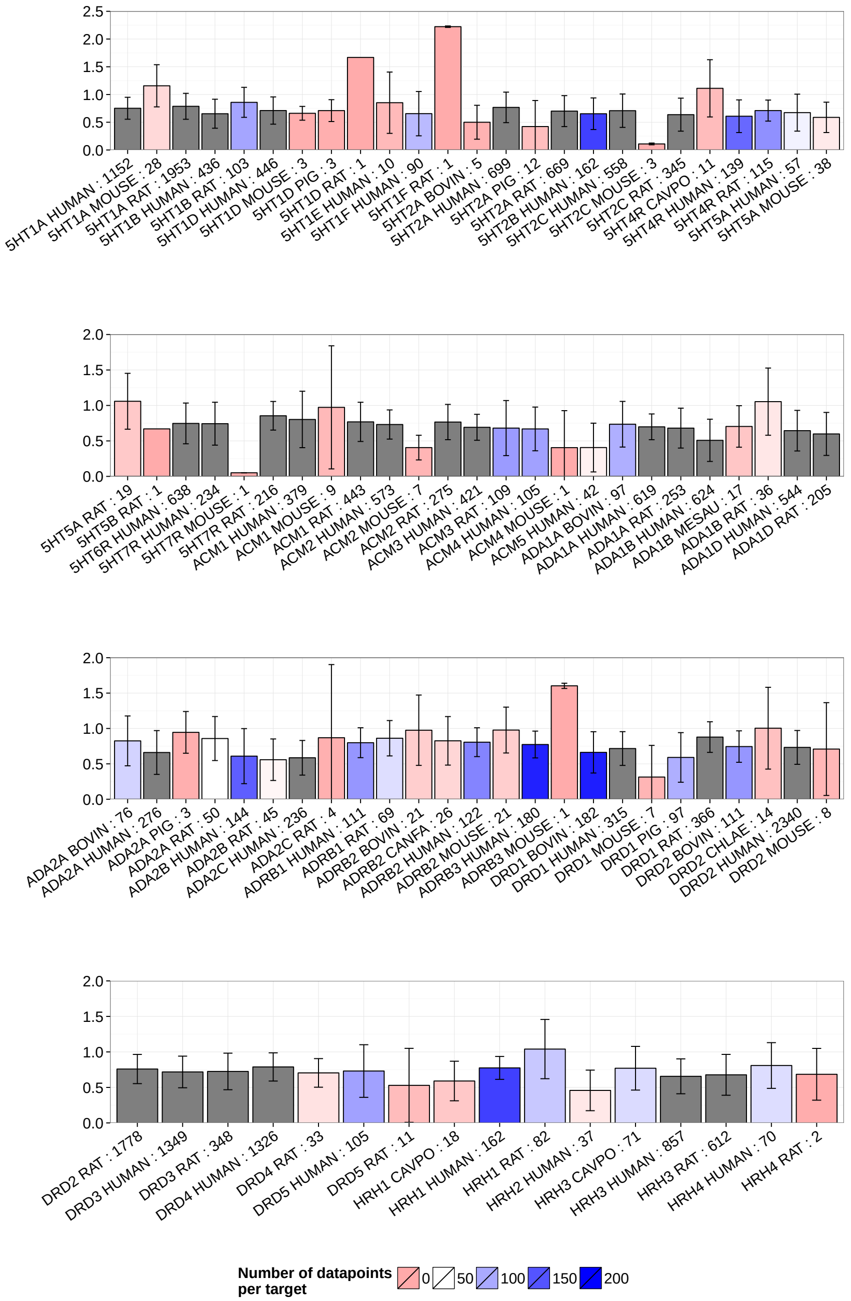

Supplementary Material, Figure S4: Evaluation of model performance per target on the GPCRs dataset. $RMSEP_{ext}$ values, averaged on ten models trained on different re-samples of the dataset, are represented by bars, colored according to the number of datapoints per target. The standard deviations on $RMSEP_{ext}$ are shown as error bars. Dark grey bars correspond to targets with more than two hundred annotated compounds.

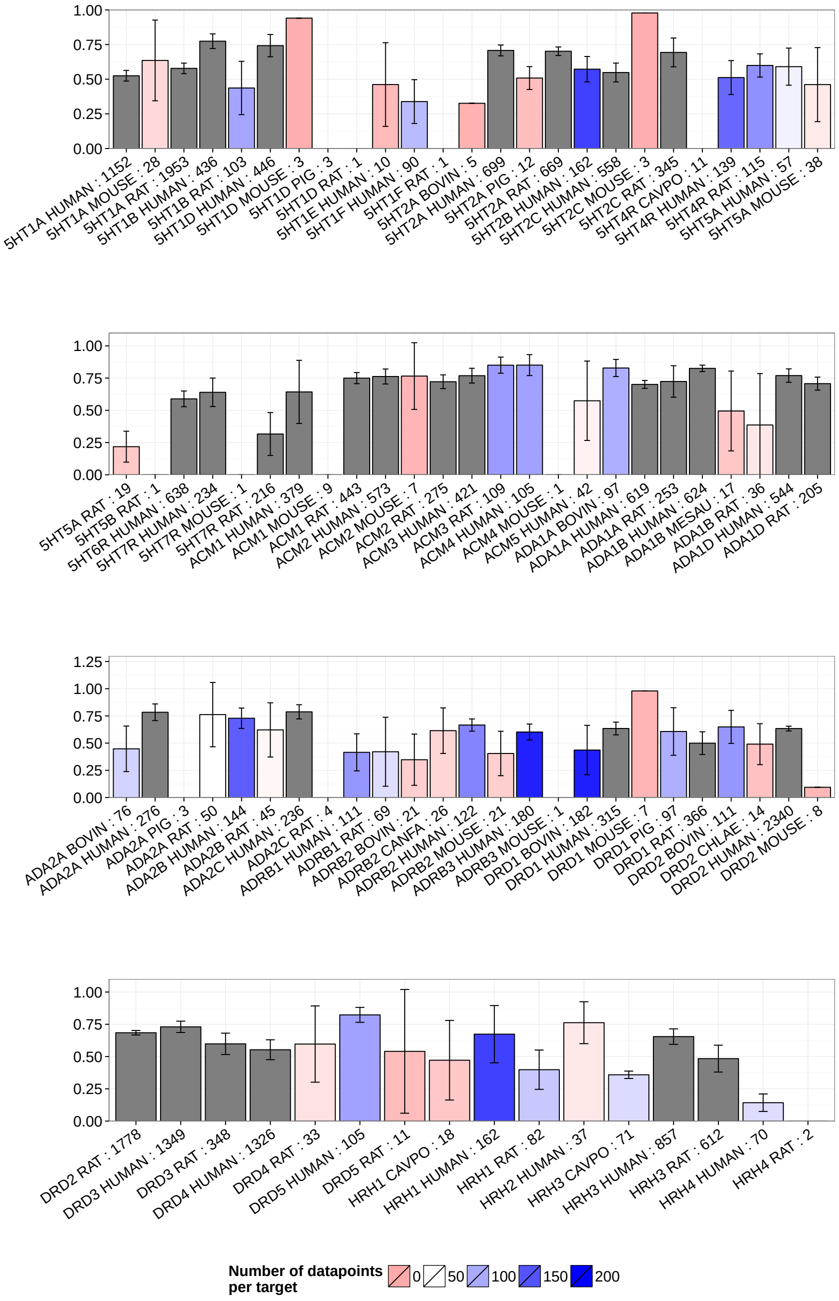

Supplementary Material, Figure S5: Evaluation of model performance per target for GPCRs dataset on the external set. R_0^2 values, averaged on ten models trained on different re-samples of the dataset, are represented by bars, colored according to the number of datapoints per target. Dark grey bars correspond to targets with more than two hundred annotated compounds. Both negative and infinite $R_{0\ ext}^2$ values were set to zero.

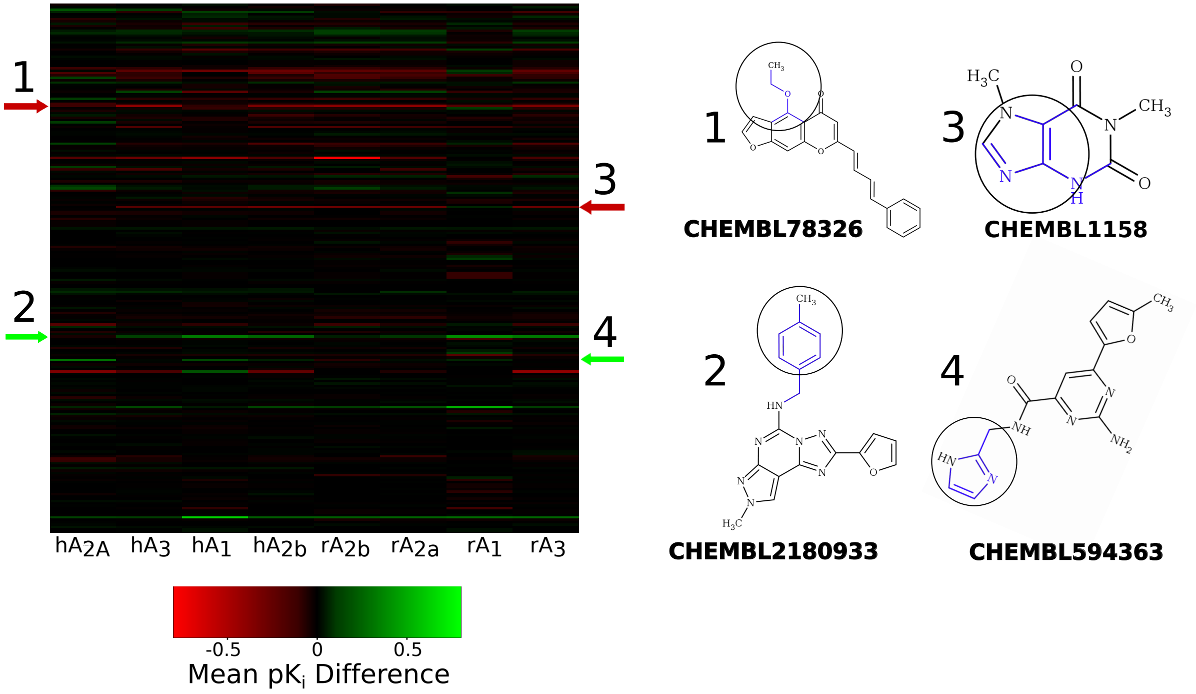

Supplementary Material, Figure S6: Heatmap representing the contribution of each chemical substructure to compounds bioactivity on each adenosine receptor. Columns are indexed by targets and rows by compound substructures. Depicted are some examples of compounds containing features beneficial (green) or deleterious (red) for bioactivity. Although a few substructures are predicted to have a beneficial or deleterious influence on the pK_i, there are others for which the effect depends on the target considered or on the rest of substructures present in a given compound (see main text). Therefore, over 90% of the substructures (black) are not implicated in compound bioactivity or their contribution depends on the other substructures present in a given compound.

Supplementary Tables

Table S1: Covariance functions (*kernels*) formula.

Bessel Kernel	$K(x_j, x_k) = -Bessel^{n}_{(nu+1)}(\sigma\lvert x_j - x_k\rvert^2)$
Laplacian Kernel	$K(x_j, x_k) = e^{-\sigma\lvert\lvert x_j - x_k\rvert\rvert}$
NP Kernel	$K(x_j, x_k) = \frac{scale\ x_j^T x_k + offset^{degree}}{\sqrt{xx^T yy^T}}$
Polynomial Kernel	$K(x_j, x_k) = scale\ x_j^T x_k + offset^{degree}$
PUK Kernel	$K(x_j, x_k) = \frac{1}{[1+(\frac{2\sqrt{\lvert\lvert x_j - x_k\rvert\rvert^2\ \sqrt{2^{(\frac{1}{\omega})}-1}}}{\sigma})^2]^{\omega}}$
Radial Kernel	$K(x_j, x_k) = e^{-\frac{\lvert\lvert x_j - x_k\rvert\rvert^2}{2l^2}}$

Table S2: Number of datapoints per GPCR. Those receptors highlighted by a '*' symbol correspond to those present in a subset of human GPCRs which was first modeled with GP (see subsection GP performance per Target). GPCRs are named according to UniProtKB/ Swiss-Prot database.[7]

Protein ID	Frequency	Protein ID	Frequency	Protein ID	Frequency
5HT1A HUMAN*	1152	ACM1 HUMAN*	379	ADRB2 HUMAN*	122
5HT1A MOUSE	28	ACM1 MOUSE	9	ADRB2 MOUSE	21
5HT1A RAT	1953	ACM1 RAT	443	ADRB3 HUMAN*	190
5HT1B HUMAN*	436	ACM2 HUMAN*	573	ADRB3 MOUSE	1
5HT1B RAT	103	ACM2 MOUSE	7	DRD1 BOVIN	182
5HT1D HUMAN*	446	ACM2 RAT	275	DRD1 HUMAN*	315
5HT1D MOUSE	5	ACM3 HUMAN*	421	DRD1 MOUSE	7
5HT1D PIG	3	ACM3 RAT	109	DRD1 PIG	97
5HT1D RAT	1	ACM4 HUMAN	105	DRD1 RAT	366
5HT1E HUMAN	10	ACM4 MOUSE	1	DRD2 BOVIN	111
5HT1F HUMAN	90	ACM5 HUMAN	42	DRD2 CHLAE	14
5HT1F RAT	1	ADA1A BOVIN	97	DRD2 HUMAN*	2340
5HT2A BOVIN	5	ADA1A HUMAN*	619	DRD2 MOUSE	8
5HT2A HUMAN*	699	ADA1A RAT	253	DRD2 RAT	1778
5HT2A PIG	12	ADA1B HUMAN*	624	DRD3 HUMAN*	1349
5HT2A RAT	669	ADA1B MESAU	17	DRD3 RAT	348
5HT2B HUMAN*	162	ADA1B RAT	36	DRD4 HUMAN*	1326
5HT2C HUMAN*	558	ADA1D HUMAN	544	DRD4 RAT	35
5HT2C MOUSE	3	ADA1D RAT	205	DRD5 HUMAN*	134
5HT2C RAT	345	ADA2A BOVIN	76	DRD5 RAT	11
5HT4R CAVPO	11	ADA2A HUMAN*	276	HRH1 CAVPO	18
5HT4R HUMAN	139	ADA2A PIG	3	HRH1 HUMAN*	162
5HT4R RAT	115	ADA2A RAT	50	HRH1 RAT	82
5HT5A HUMAN	57	ADA2B HUMAN	144	HRH2 HUMAN	37
5HT5A MOUSE	38	ADA2B RAT	45	HRH3 CAVPO	73
5HT5A RAT	19	ADA2C HUMAN*	236	HRH3 HUMAN*	857
5HT5B RAT	1	ADA2C RAT	4	HRH3 RAT	612
5HT6R HUMAN*	638	ADRB1 HUMAN*	111	HRH4 HUMAN*	70
5HT7R HUMAN*	234	ADRB1 RAT	69	HRH4 RAT	2
5HT7R MOUSE	1	ADRB2 BOVIN	21		
5HT7R RAT	216	ADRB2 CANFA	26		

Table S3: Internal and external validation metrics for the PCM models.

Adenosine Receptors Dataset

	R^2_{int}	$\text{RMSEP}_{\text{int}}$	R^2_{ext}	$R^2_{0\ ext}$	k'	Q^2_{ext}	$\text{RMSEP}_{\text{ext}}$	$(R^2_{ext} - R^2_{0\ ext})/R^2_{ext}$	Variance
GP Bessel	0.64	0.70	0.71	0.70	1.00	0.70	0.63	0.00	0.29
GP Laplacian	0.67	0.68	0.68	0.67	1.01	0.67	0.66	0.07	0.29
GP Norm. Polynomial (NP)	0.69	0.65	0.75	0.75	0.99	0.74	0.58	0.00	0.29
GP Polynomial	0.70	0.64	0.71	0.70	1.00	0.70	0.63	0.01	0.29
GP PUK	0.57	0.79	0.59	0.56	1.01	0.56	0.77	0.05	0.29
GP Radial	0.65	0.69	0.66	0.65	1.00	0.65	0.68	0.01	0.29
PLS	0.29	0.97	0.30	0.30	1.00	0.30	1.00	0.00	-
SVM Norm. Polynomial (NP)	0.70	0.64	0.73	0.73	1.00	0.73	0.60	0.00	-
SVM Polynomial	0.71	0.63	0.71	0.71	1.00	0.71	0.62	0.00	-
SVM Radial	0.68	0.65	0.70	0.70	1.00	0.70	0.64	0.01	-
QSAR	0.31	0.70	0.31	0.31	1.01	0.31	0.96	0.00	0.29

Aminergic GPCRs Dataset

	R^2_{int}	$\text{RMSEP}_{\text{int}}$	R^2_{ext}	$R^2_{0\ ext}$	k'	Q^2_{ext}	$\text{RMSEP}_{\text{ext}}$	$(R^2_{ext} - R^2_{0\ ext})/R^2_{ext}$	Variance
GP Bessel	0.56	0.83	0.58	0.56	1.00	0.57	0.80	0.04	0.29
GP Laplacian	0.62	0.78	0.64	0.63	1.00	0.63	0.75	0.02	0.29
GP Norm. Polynomial (NP)	0.69	0.68	0.73	0.72	0.10	0.72	0.66	0.01	0.29
GP Polynomial	0.68	0.71	0.70	0.70	1.00	0.70	0.68	0.00	0.29
GP PUK	0.46	0.93	0.49	0.46	1.00	0.46	0.90	0.05	0.29
GP Radial	0.69	0.69	0.72	0.71	1.00	0.71	0.66	0.01	0.29
PLS	0.69	0.69	0.27	0.27	1.00	0.27	1.05	0.00	-
SVM Norm. Polynomial (NP)	0.69	0.68	0.72	0.72	1.00	0.72	0.66	0.00	-
SVM Polynomial	0.69	0.69	0.71	0.71	1.00	0.71	0.66	0.00	-
SVM Radial	0.69	0.69	0.72	0.72	1.00	0.72	0.66	0.00	-
QSAR	0.38	0.98	0.38	0.38	1.01	0.37	0.97	0.01	0.29

Dengue virus NS3 proteases Dataset

	R^2_{int}	$\text{RMSEP}_{\text{int}}$	R^2_{ext}	$R^2_{0\ ext}$	k'	Q^2_{ext}	$\text{RMSEP}_{\text{ext}}$	$(R^2_{ext} - R^2_{0\ ext})/R^2_{ext}$	Variance
GP Bessel	0.91	0.43	0.92	0.92	0.98	0.92	0.44	0.00	0.07
GP Laplacian	0.88	0.54	0.92	0.91	1.16	0.89	0.50	0.00	0.07
GP Linear	0.91	0.45	0.92	0.91	0.92	0.90	0.48	0.01	0.07
GP Norm. Polynomial (NP)	0.88	0.50	0.92	0.91	0.99	0.90	0.48	0.02	0.07
GP Polynomial	0.91	0.42	0.92	0.92	0.98	0.92	0.44	0.00	0.07
GP PUK	0.77	1.10	0.82	0.81	3.03	0.45	1.13	0.01	0.07
GP Radial	0.91	0.45	0.92	0.91	1.01	0.91	0.45	0.00	0.07
PLS	0.90	0.45	0.91	0.91	0.91	0.90	0.49	0.00	-
SVM Norm. Polynomial (NP)	0.86	0.54	0.92	0.91	0.96	0.91	0.46	0.01	-
SVM Polynomial	0.89	0.46	0.91	0.90	0.90	0.89	0.51	0.01	-
SVM Radial	0.90	0.48	0.91	0.90	1.05	0.90	0.48	0.00	-
QSAR	0.29	1.19	0.51	0.48	1.37	0.45	1.13	0.06	0.07

Abbreviations. RMSEP: root mean square error in prediction; Ext. : external; Norm: Normalized.

References

(1) Prusis, P.; Lapins, M.; Yahorava, S.; Petrovska, R.; Niyomrattanakit, P.; Katzenmeier, G.; Wikberg, J. E. S. Proteochemometrics analysis of substrate interactions with dengue virus NS3 proteases. *Bioorg. Med. Chem.* **2008**, *16*, 9369–9377.

(2) Hawkins, D. M.; Basak, S. C.; Mills, D. Assessing model fit by cross-validation. *J. Chem. Inf. Comput. Sci.* **2003**, *43*, 579–586.

(3) Consonni, V.; Ballabio, D.; Todeschini, R. Evaluation of model predictive ability by external validation techniques. *J. Chemometrics* **2010**, *24*, 194201.

(4) Golbraikh, A.; Tropsha, A. Beware of q2! *J. Mol. Graphics Modell.* **2002**, *20*, 269–276.

(5) Tropsha, A.; Golbraikh, A. Predictive Quantitative Structure-Activity Relationships Modeling. *Handbook of Chemoinformatics Algorithms* **2010**, *33*, 211.

(6) Tropsha, A.; Gramatica, P.; Gombar, V. K. The importance of being earnest: validation is the absolute essential for successful application and interpretation of QSPR models. *QSAR Comb. Sci.* **2003**, *22*, 6977.

(7) Magrane, M.; Consortium, U. UniProt Knowledgebase: a hub of integrated protein data. *Database* **2011**, *2011*.
